# The validation of measured and self-reported sleep duration and perceived sleep quality: an empirical study with three generations of smartwatches

**DOI:** 10.3389/fpsyt.2026.1767216

**Published:** 2026-04-20

**Authors:** Christina T. Saliba, Angelina R. Wilton, Katharine Sheffield, Quantia Wilkes, Miriam Anacker, Colin P. West, Paul E. Croarkin, Liselotte N. Dyrbye, Sherry S. Chesak, William V. Bobo, Arjun P. Athreya, Mohit Chauhan

**Affiliations:** 1 Department of Molecular Pharmacology and Experimental Therapeutics, Mayo Clinic, Rochester, MN, United States; 2 Center for Individualized Medicine, Mayo Clinic, Rochester, MN, United States; 3 Department of Nursing, Mayo Clinic, Jacksonville, FL, United States; 4 Center for Individualized Medicine, Mayo Clinic, Jacksonville, FL, United States; 5 Department of Medicine, Mayo Clinic, Rochester, MN, United States; 6 Department of Quantitative Health Sciences, Mayo Clinic, Rochester, MN, United States; 7 Department of Psychiatry and Psychology, Mayo Clinic, Rochester, MN, United States; 8 Department of Medicine, University of Colorado School of Medicine, Denver, CO, United States; 9 Department of Nursing, Mayo Clinic, Rochester, MN, United States; 10 School of Nursing, University of Minnesota, Minneapolis-St. Paul, MN, United States; 11 Department of Behavioral Sciences and Social Medicine, Florida State University, Tallahassee, FL, United States; 12 Department of Psychiatry and Psychology, Mayo Clinic, Jacksonville, FL, United States

**Keywords:** empirical validation, shift workers, sleep, sleep quality, wearables

## Abstract

**Introduction:**

Sleep is routinely assessed in the management of mental health conditions. Wearable technologies like smartwatches offer a non-intrusive method to quantitatively measure sleep. However, there are limited empirical benchmarks for sleep duration and sleep quality measured by wearables against user reports. This study aims to evaluate the concordance between user-reported and smartwatch-measured hours of sleep and sleep quality.

**Methods:**

Participants were recruited from two decentralized digital health well-being studies and completed a 7-day sleep diary while simultaneously wearing their smartwatch to sleep (November 7, 2023 - June 30, 2024). Participants self-reported sleep timestamps and perceived sleep quality using the Sleep Quality Scale. Sleep timestamps and quality were also derived from their smartwatches (Garmin Vívoactive 4 2019, Garmin Venu 2 Plus 2022, and Garmin Venu 3/3S 2023). Statistical analyses included paired t-tests, equipercentile linking, and chi-square tests to assess agreement between smartwatch and self-reported sleep parameters. Exploratory analyses established the difference between reported and recorded sleep duration in healthcare shift workers.

**Results:**

From 841 sleep instances reported by 130 participants wearing three different generation smartwatches, the mean difference in sleep duration between smartwatch-recorded and participant-reported was 21.22 (Garmin Vívoactive), 11.67 (Garmin Venu 2 Plus), and 6.58 (Garmin Venu 3/3S) minutes, respectively. There were statistically significant between-group differences in mean sleep durations assessed by participant self-report vs. Vívoactive 4 smartwatches, but not self-report vs. Venu 2 Plus or Venu 3/3S smartwatches. Equipercentile linking revealed concordance between smartwatch sleep scores and self-reported sleep quality using the Sleep Quality Scale (SQS) between 4 and 7, with disagreements observed at the SQS ranges from 0–4 and 7-10.

**Conclusions:**

These results suggest that wearables can reliably measure sleep duration, and future research warrants improvements in algorithms that estimate sleep quality with validations across different wearable vendors.

## Introduction

High-quality sleep is a key contributor to mental and physical health, emotional wellbeing, and daily functioning (1–4). Wearable consumer technologies such as fitness trackers and smartwatches have become popular self-tracking tools for individuals motivated to monitor and improve their sleep habits (5, 6). These devices are user-friendly and offer a cost-effective way to collect data on sleep and other physiological measurements such as heart rate, steps, and body temperature (7, 8). The longitudinal monitoring of sleep parameters with commercial-grade wearables (e.g., smartwatches) in real-world settings (including in individuals with diurnal rhythms such as night-shift workers) provides an opportunity to detect the association of sleep patterns with health outcomes (9, 10).

Several recent population-scale cohort studies have utilized smartwatch data to advance discoveries of mental health across the lifespan. In children and adolescents, investigators in the Adolescent Brain Cognitive Development (ABCD) Study have utilized data from Fitbit smartwatches to identify biomarkers of sleep and activity to diagnose and predict neurodevelopment and behavioral problems in adolescents (11–15). In adults, the smartwatch data from participants in United Kingdom’s biobank and the All-of-Us program in the United States has been used to identify biomarkers relating to sleep associated with mental health and other chronic conditions (16, 17).

With the growing adoption of commercial-grade wearables in mental health research, validating smartwatch-derived sleep estimates against self-report is important for several reasons. Discrepancies between subjective and objective measures may reflect clinically relevant conditions such as sleep misperception. Additionally, the directionality of the difference (i.e., overestimated sleep or underestimated sleep against user reported hours) may inform how measurement errors are to be adjusted in analytical formulations such as Artificial Intelligence (AI) that use sleep data in estimating human functioning. Understanding whether newer device generations reduce such discrepancies helps determine whether algorithmic refinements are closing the gap between measurement and lived experience. Lastly, as sleep and its quality serve as indicators of one’s functioning (18), these devices are also suitable for large-scale real-world deployment to capture sleep data over extended monitoring periods that potentially enables digital health interventions modeling. Smartwatches’ ability to passively capture sleep data supports the development of scalable digital health interventions, enabling earlier detection of health conditions using sleep (or associated measures including sleep stages) as a biomarker and offering more personalized, data-driven care (19). To this end, there have been efforts in the ABCD study to establish concordance in smartwatch-derived sleep measurements in adolescent participants and their caregivers (20). However, few studies have established the concordance between self-reported and wearable-measured sleep duration and quality (21, 22). Therefore, there is a knowledge gap regarding empirical differences in smartwatch-derived sleep data (e.g., duration, sleep quality) and those reported by users (including those with diurnal sleep patterns) in real-world settings.

**Figure 1:**
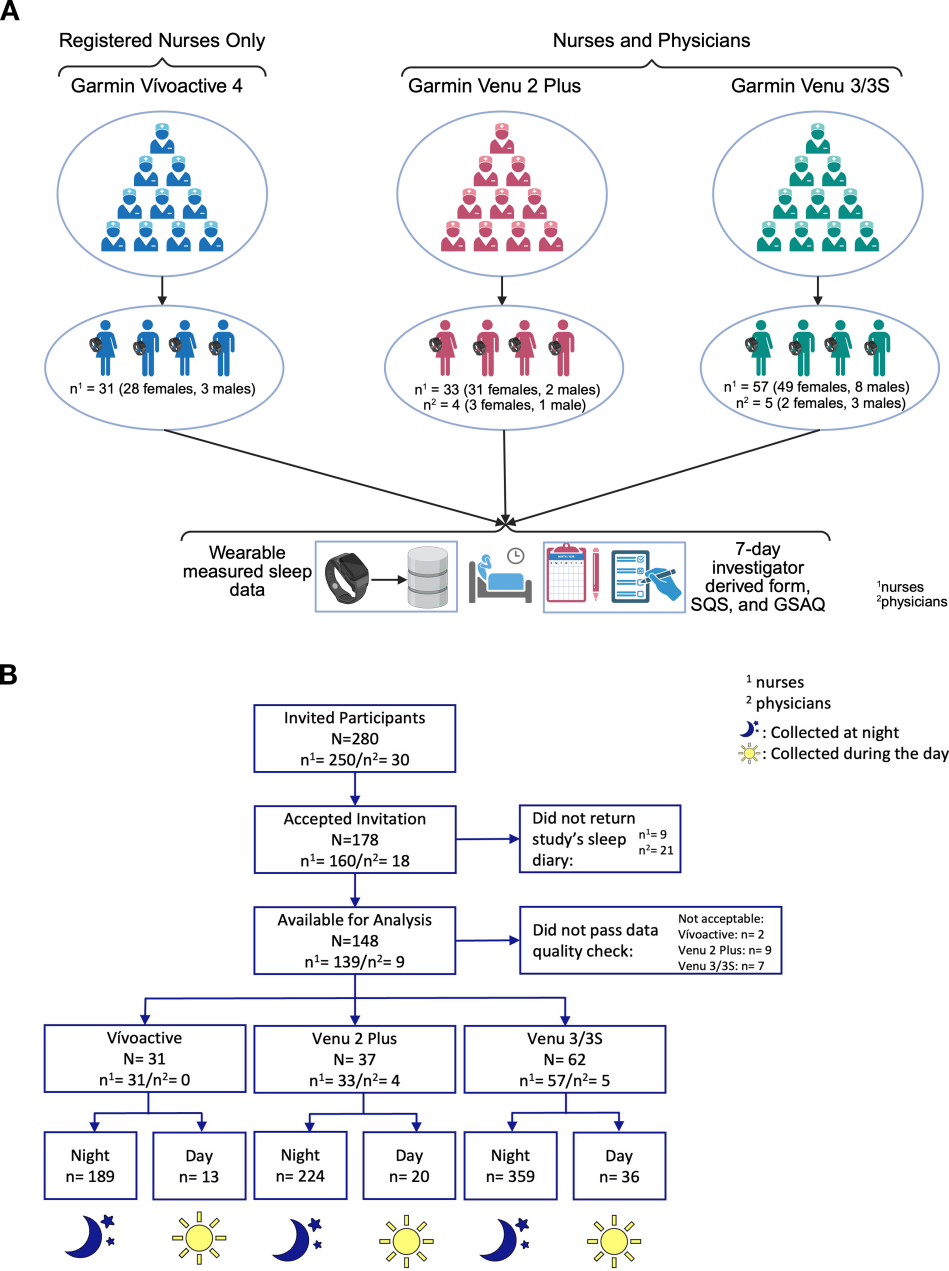
**(A)** Study recruitment overview. **(B)** Study flow diagram for all three devices.

This study addresses the aforementioned knowledge gaps by using prospectively collected sleep diaries from participants who were issued smartwatches in ongoing healthcare professional well-being studies at the Mayo Clinic (see **Figure 1A**). Participants were asked to report 7 instances of sleep (either during the day or night based on their shift-type) with timestamps of when they went to sleep and woke up, and their perceived quality of sleep. These user-reported measurements were validated against measured data from smartwatches. This study hypothesized that newer generations and models of smartwatches would show enhanced agreement in measuring sleep duration, while discrepancies in sleep quality assessment would persist due to the interpretive complexity involved and the proprietary algorithms created by the smartwatch companies.

## Methods and materials

### Study participants

Participants were recruited from two separate parent studies on wellbeing of healthcare professionals [registered nurses (RNs): NCT05481138, accrued 531 RNs; physicians: NCT05463250, accrued 81 physicians] employed at Mayo Clinic’s Minnesota and Florida sites. Participating RNs and physicians in these two studies are adults aged between 20–70 years of age and categorized as either day-, night- or flex (the ability to work either day or night shifts) shift professionals. In these two studies, participating RNs and physicians were expected to wear a smartwatch (issued by the study) for 6 to 12 months for ≥70% of time during a 24-hour period (23). Participants were not excluded in the parent wellbeing studies based on any preexisting health conditions. This sleep validation study was advertised in an email to participants of the two burnout studies who wore the study smartwatch >50% of the time for at least one study quarter. Participants for this sleep validation study were considered enrolled after receiving their electronic consent to the study procedures approved by the Mayo Clinic Institutional Review Board. Given the lack of prior measurements or evidence of power and detection levels in sleep measures, sample size estimates were not derived.

### Decentralized study procedures

After providing consent, study participants received an investigator-developed sleep diary (see **Supplementary Attachment 1**), the Sleep Quality Scale (SQS) (24) and the Global Sleep Assessment Questionnaire (GSAQ) (25) delivered by email or in a concealed envelope based on their preference. The sleep diary recorded sleep duration and quality of sleep for 7 instances of sleeping, either during the night or during the day to accommodate shiftwork schedules. Upon completing the diary and GSAQ, participants were requested to either send the completed documents via email (scanned or photographed), leave the sealed envelope at a specified location to be picked up by one of the study team members, or return the documents through a confidential, intra-campus mail service. Participants were remunerated $5 per instance of sleep data provided, with a maximum of $35 for all 7 instances.

### Study smartwatches and smartwatch data collection

Garmin smartwatches were issued to participants in the parent studies as they were compatible with both Apple and Android smartphone ecosystems. RNs received either Garmin Vívoactive 4 released in 2019 (if accrued in October 2022), or Garmin Venu 2 Plus released in 2022 (if accrued in April 2023), or Garmin Venu 3/3S released in 2023 (if accrued in January 2024). Physicians received either Garmin Venu 2 Plus (if smartwatch issued in July 2023) or Garmin Venu 3/3S (if smartwatch issued in Dec 2024). One cohort of RNs who received the Garmin Vívoactive 4 (for the first 12 months in the study) were also issued Garmin Venu 3/3S if they continued to participate in the burnout study for a second 12-month period. The data from all participants’ smartwatches were routinely collected through Fitabase (by Small Steps Labs) for study analyses, and participants had access to their data (including all other smartwatch measures such as heartrate, sleep, activity) through the Garmin Connect mobile application (see **Figure 1A**).

### Study measures

#### Sociodemographic and shift type

Age (in years), sex at birth (male/female), race (American Indian/Alaska Native, Asian, Black/African American, Native Hawaiian or Other Pacific Islander, White), and ethnicity (Hispanic or Latino/Not Hispanic or Latino) comprised the sociodemographic measures. Participants were subclassified based on shift types: day, night, and flex (i.e., those who work both day and night shifts based on shift availability).

#### Global sleep assessment questionnaire

The Global Sleep Assessment Questionnaire (GSAQ) (25) is a self-administered tool designed and validated to screen for a broad range of sleep disorders. The GSAQ used in this study was an 11-item self-administered screening tool that uses four response options (never, sometimes, usually, and always). The GSAQ is designed to screen multiple sleep disorders including primary insomnia, insomnia associated with mental disorder (IME), obstructive sleep apnea (OSA), restless legs syndrome (RLS), periodic limb movement (PLM), parasomnias, and shift work sleep disorder. This tool was administered to assess the impact of differences in duration of sleep based on items relating to sleep disorders.

#### Participant-reported sleep timestamps

The sleep diary (**Supplementary Material 1**) collected 7 instances of self-reported timestamp of sleep in the following format: the date going to sleep (format: MM/DD/YYYY), time going to sleep (format: HH/MIN AM/PM), date waking up after sleep (format: MM/DD/YYYY), and time waking up after sleep (format: HH/MIN AM/PM). Participants were asked to report the closest approximate times they went to bed and woke up. They were specifically asked to avoid reporting the timestamps by looking at the bed- and awake time from Garmin Connect app or the smartwatch.

#### Sleep quality

For each sleep instance, participants completed the validated single item Sleep Quality Scale (SQS), that records sleep quality on a 0-10-point scale: Terrible (0), Poor (1-3), Fair (4-6), Good (7-9), and Excellent (10) (24).

#### Smartwatch generated measures

All three Garmin smartwatches provided the time a participant was estimated to begin and end sleep, which were obtained from Fitabase for the date and times participants provided their sleep timestamps. The Garmin Venu 2 Plus and Venu 3/3S smartwatches also provided continuous sleep quality scores (ranging from 0 to 100) and categorical sleep quality ratings (poor, fair, good, and excellent) using proprietary algorithms. The categorical sleep quality ratings were based on smartwatch-estimated continuous sleep quality scores as follows: excellent (90 – 100), good (80 – 89), fair (60 – 79), and poor (< 60) (26). Participants with Garmin Venu 2 Plus and Venu 3/3S smartwatches were also asked to report the Garmin-generated sleep score and sleep quality in the sleep diary (as reported in the Garmin Connect app that summarizes smartwatch data for users).

### Data preprocessing

The study team verified the reported and the measured sleep times to the minute for all 7 instances, by comparing the smartwatch data downloaded from Fitabase to the reported data from the participant. If the difference between the reported and the measured sleep duration was the same (i.e., 0 minutes) for all 7 instances, a study team member asked participant if they self-reported their sleep time from either the Garmin Connect App or the smartwatch in their diary. In such instances, participants were requested to complete a sleep diary providing sleep times without looking at the watch or the app. If participants chose not to provide a new sleep diary, the original data with 0-minute difference in reported and measured sleep was excluded from the analysis.

### Statistical analysis

Descriptive statistics (Chi-square test and t-tests) were used to characterize the sociodemographic variables and shift type. Sleep instances were excluded from the study if self-reported sleep times (in the diary) were the same (to the precise minute) as the timestamp reported in the smartwatch data, reflecting a likely chance of a participant reporting sleep times from the smartwatch or the Garmin Connect app as opposed to reporting their approximate sleep time.

Frequencies of overestimation (i.e., smartwatch detects a longer duration of sleep than reported by participant) or underestimation (i.e., smartwatch detects a shorter duration of sleep than reported by participant) of sleep are also reported for each watch type and by shift-type. Frequencies of overestimation and underestimation were compared for each smartwatch model using chi-square tests to determine whether one occurred more often than the other.

Equipercentile linking was used to equate participant-reported SQS scores with smartwatch-generated sleep scores. Equipercentile linking is a non-parametric statistical process that is used to find equivalent points on separate but correlated scales (e.g., the SQS and smartwatch score scale), and accounts for possible measurement error for each of the scales. Chi-square tests were used to compare participant quality of sleep (based on SQS score mapping) and Garmin-reported quality of sleep.

Multi-way analyses of variance (ANOVAs) were conducted to examine the relationship between GSAQ item responses (Q1 through Q11) and the difference in average sleep duration between smartwatch data and participant-reported sleep diaries (measured in minutes). Each model controlled for the effects of age and sex. All analyses were conducted using R (27)(Version 4.4.3).

## Results

### Participant characteristics

A total of 250 RNs (out of 531) and 30 physicians (out of 81) who participated in the parent burnout studies were invited to participate in this sleep validation study (see flow diagram in **Figure 1B**). Among those invited, 178 (160 RNs; 18 physicians) provided informed consent, and 130 (121 RNs; 9 physicians) of whom completed the study (see flow diagram in **Figure 1B**) with data available for analyses. The sociodemographic and shift type characteristics of the enrolled participants are reported in **Table 1**.

**Table 1:** Demographics.

Variables and measures	Overall (n=130)	Vívoactive 4 (n=31)	Venu 2 Plus (n=37)	Venu 3/3S (n=62)	^1^p-value
Age in Years, mean (SD)	38.3 (10.9)	37.9 (10.1)	41.6 (11.6)	36.6 (10.6)	0.09
Sex at Birth					0.35
Female	113 (86.9%)	28 (90.3%)	34 (91.9%)	51 (82.3%)	
Male	17 (13.1%)	3 (8.7%)	3 (8.1%)	11 (17.7%)	
Race					0.15
White	108 (83%)	28 (90.3%)	25 (67.6%)	55 (88.7%)	
Black/African American	4 (3.1%)	1 (3.2%)	2 (5.4%)	1 (1.6%)	
Asian	15 (11.5%)	2 (6.5%)	9 (24.3%)	4 (6.5%)	
American Indian/Alaska Native	1 (0.8%)	0	0	1 (1.6%)	
Native Hawaiian or Other Pacific Islander	1 (0.8%)	0	0	1 (1.6%)	
Unknown	1 (0.8%)	0	1 (2.7%)	0	
Ethnicity					0.79
Hispanic or Latino	6 (4.6%)	1 (3.2%)	3 (8.1%)	2 (3.2%)	
Not Hispanic or Latino	121 (93.1%)	29 (93.6%)	33 (89.2%)	59 (95.2%)	
Unknown	3 (2.3%)	1 (3.2%)	1 (2.7%)	1 (1.6%)	
Shift Type^2^					0.65
100% Day Shift	73 (56.2%)	21 (67.7%)	20 (54.1%)	32 (51.6%)	
100% Night Shift	20 (15.4%)	3 (9.7%)	5 (13.5%)	12 (19.4%)	
Flex Shift	34 (26.1%)	7 (22.6%)	10 (27%)	17 (27.4%)	
Unknown	3 (2.3%)	0	2 (5.4%)	1 (1.6%)	

^1^Pearson’s Chi-squared test, Fisher’s exact test, One-Way ANOVA.

^2^Shift Type recorded in registered nurses only.

Among the accrued participants, 31 RNs wore the Garmin Vívoactive 4, 37 participants (33 RNs; 4 physicians) wore the Garmin Venu 2 Plus, and 62 participants (57 RNs; 5 physicians) wore the Garmin Venu 3/3S smartwatches. Fourteen of the 57 RNs who provided data for the Garmin Vívoactive 4 (between October 2022-2023) also participated in the sleep validation study while they wore the Garmin Venu 3/3S smartwatch (between October 2023-2024).

Among the 130 participants, 953 instances of sleep were reported in the sleep diaries. The excluded participants all had all 7 instances of sleep reported that matched with the smartwatch measurements to the exact minute and were hence excluded. In the final data available for analyses, 772 of 841 sleep instances were during the night hours and the remaining 69 were during the day.

### Difference in participant-reported vs. smartwatch-measured sleep durations

**Figure 2:**
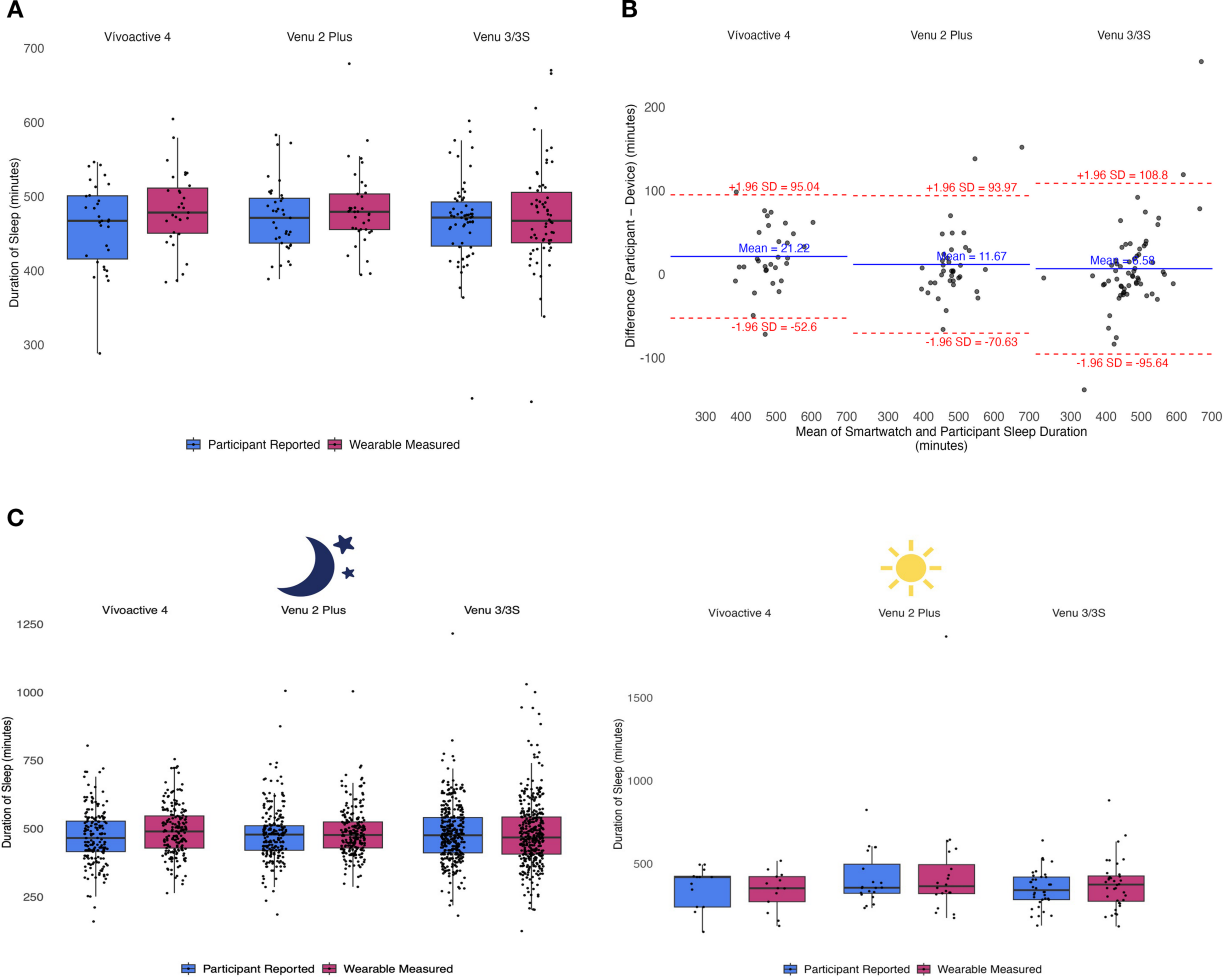
**(A)** Results of paired t-test comparing reported versus measured sleep by device **(B)** Bland-Altman plot for sleep duration Garmin vs participant diary **(C)** Results of paired t-test comparing reported versus measured sleep by device by time of day.

The difference in sleep duration (measured by smartwatch – self-reported) between that measured by the smartwatch and self-reported duration are tabulated in **Table 2** and illustrated in **Figures 2A, B**. The sleep duration measured by the Vívoactive 4 (N = 31) smartwatch was significantly greater than self-reported sleep duration (difference = +21.22 minutes, t(30) = 3.14, *P* = .004, 95% CI [7, 35]). The sleep duration measured by the Venu 2 Plus (N = 37) smartwatch was numerically greater than self-reported duration, but not statistically significant than self-reported sleep duration (difference = +11.67 minutes, t(36) = 1.69, *P* = .10, 95% CI [-2, 26]). The sleep duration measured by Venu the 3/3S (N = 62) smartwatch was greater, but not statistically significant than self-reported sleep duration (difference = +6.58 minutes, t(61) = .99, *P* = .32, 95% CI [-7, 20]).

**Table 2:** Results of paired t-test comparing average reported sleep versus measured sleep by device and by time of day.

Device and sleep time	Mean difference in sleep duration (in minutes; measured-reported)	p-value	t	df	CI (minutes)	
					Lower	Upper
Vívoactive 4 (n=31)	+21.22	0.004	3.14	30	7	35
Venu 2 Plus (n=37)	+11.67	0.1	1.69	36	-2	26
Venu 3/3S (n=62)	+6.58	0.32	0.99	61	-7	20
Vívoactive 4						
Night (n=189)	+22	< 0.001	5.07	188	14	31
Day (n=13)	-18	0.55	-0.61	12	-82	46
Venu 2 Plus						
Night (n=224)	8	0.1	1.63	223	-2	17
Day (n=20)	47	0.39	1.54	19	-67	161
Venu 3/3S						
Night (n=359)	-0.5	0.92	-0.98	358	-10	9
Day (n=36)	28	0.13	1.54	35	-9	65

For each smartwatch model, number of sleep instances in which the smartwatch overestimated versus underestimated sleep duration was compared using chi-square tests. For the Vívoactive 4, the frequency of overestimation of total sleep duration was statistically significant (P = .004), with more episodes of overestimation than underestimation of total sleep duration (see **Supplementary Figure 1**). However, for the Venu 2 Plus and the Venu 3/3S, the frequency of overestimation or underestimation were not statistically significant (P = .10 for Venu 2 Plus and P = .32 for Venu 3/3S, respectively; see **Supplementary Figure 1**), indicating that these watch models were neither overestimating nor underestimating the duration of sleep.

### Difference in participant-reported vs. smartwatch-measured sleep durations by the time of sleep

For participants using the Vívoactive 4, the mean difference in nighttime sleep duration between wearable-measured and self-reported sleep was +22 minutes, with a statistically significant difference observed (t(188) = 5.07, *P* <.001, 95% CI [14, 31]). However, for daytime sleep the mean difference was -18 minutes (i.e., smartwatch underestimated sleep), and no statistically significant difference was found (t(12) = -.61, *P* = .55, 95% CI [-82, 46]).

For participants using the Venu 2 Plus, the mean difference in nighttime sleep duration was +8 minutes, but this was not statistically significant (t(223) = 1.63, *P* = .10, 95% CI [-2, 17]). For daytime, the mean difference was +47 minutes, and no statistically significant difference was observed (t(19) = 1.54, *P* = .39, 95% CI [-67, 161]).

For participants using the Venu 3/3S, the mean difference in nighttime sleep duration was -0.5 minutes, with no statistically significant difference detected (t(358) = -.98, *P* = .92, 95% CI [-10, 9]). For daytime sleep, the mean difference was +28 minutes and was not statistically significant (t(35) = 1.54, *P* = .13, 95% CI [-9, 65]) (see **Figure 2C**).

### Differences in quantitative assessment of sleep quality

**Figure 3:**
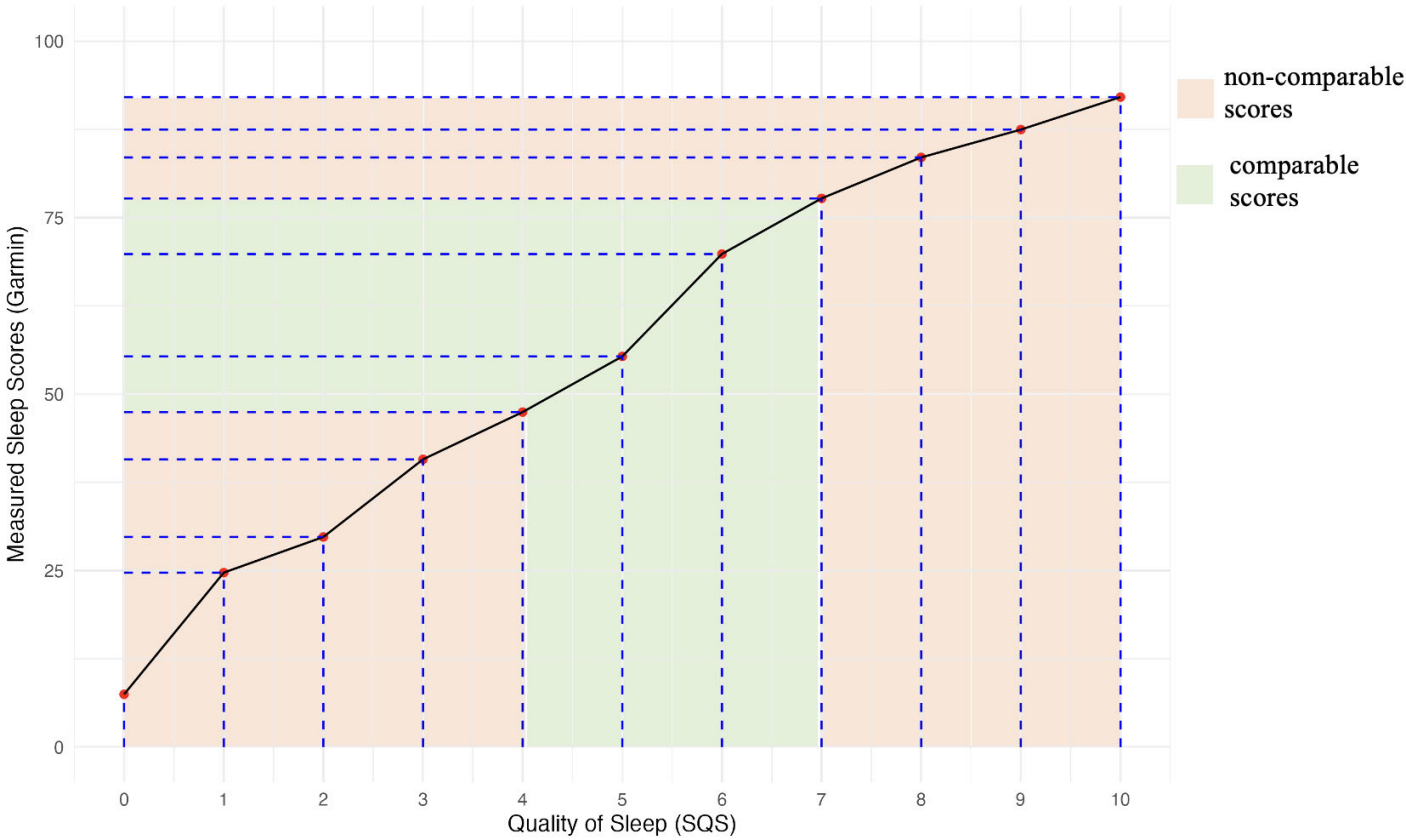
Equipercentile Matching of Quality of Sleep (SQS) and Measured Sleep Scores (Garmin).

Self-reported SQS scores were paired with smartwatch-derived sleep quality ratings for the Venu 2 Plus and Venu 3/3s pooled together using equipercentile linking (see **Figure 3**). Based on equipercentile linkage, Garmin scores below 40 corresponded to SQS scores of 3 or lower, indicating poor sleep quality. In the mid-range, Garmin scores between 47 and 78 aligned with SQS scores of 4 to 7, representing moderate sleep quality. At higher levels, Garmin scores above 83 equated to SQS scores of 8 or higher, signifying good to excellent sleep quality. The highest equated Garmin score of 92 corresponded to the maximum SQS score of 10, reflecting the upper limit of sleep quality measurement.

### Differences in assessment of sleep quality

Chi-square tests were used to examine the concordance of categorical sleep quality ratings from the SQS and from the categories defined by the Venu 2 Plus and Venu 3/3S smartwatches. There was a statistically significant difference between the SQS- and smartwatch-estimated sleep quality with both smartwatch data combined, (X^2^(20, N = 616) = 157.74, *P* <.001), with concordance only in the “Good-Good” and “Poor-Poor” classifications (see **Supplementary Table 4**). Statistically significant differences in participant-estimated vs. smartwatch-reported sleep quality were also observed for the Venu 2 Plus (X^2^(9, N = 209) = 46.01, *P* <.001) and the Venu 3/3S (X^2^(20, N = 337) = 99.5, *P* <.001) smartwatches, respectively.

### GSAQ and sleep durations

None of the individual item scores on the GSAQ were significantly associated (*P*>0.05) with differences in sleep duration, when controlling for age, sex, and device model (see **Supplementary Table 1**).

## Discussion

This study validated sleep duration and quality metrics derived from three generations of Garmin smartwatches against self-reported sleep data in a cohort of healthcare professionals enrolled in wellbeing studies using smartwatches. Across 841 reported sleep instances from 130 participants, newer smartwatch models demonstrated increasing concordance with self-reported sleep duration, suggesting progressive refinement in proprietary algorithms for measuring sleep duration. Discrepancies emerged in assessments of sleep quality, particularly at the extremes of perceived sleep experience. Future research warrant improvements in algorithms that estimate sleep quality with validations across different wearable vendors.

This study’s findings underscore the need for a nuanced approach when applying machine learning (ML) and AI algorithms to wearable-generated sleep data. As the study’s results suggest, ML/AI models must be designed with sufficient flexibility to avoid being overly sensitive to fixed cut-offs—particularly in the interpretation of sleep quality, which is inherently subjective. First, the study’s findings indicate that researchers and developers should account for model-dependent differences in sleep duration estimates, which in this study ranged from 6.58 to 21.22 minutes, as well as the possibility of systematic over- or under-estimation. Second, in relation to sleep quality, equipercentile linking analyses revealed both alignment and misalignment between participant-reported scores and device-generated outputs. For SQS values between 4 and 7, the smartwatch scores corresponded closely with self-reports, indicating consistent estimation of moderate sleep quality. However, at the lower end (SQS 0–3), devices tended to overestimate sleep quality, and at the upper end (SQS 8–10), they tended to underestimate it. These patterns point to the need for improved algorithmic sensitivity in estimating sleep quality, consistent with prior concerns raised in the digital health literature (28).

Self-report of sleep quality and sleep-related measures are often subject to bias based on comorbid health conditions and sex or gender. Prior studies have shown sleep durations and self-report of sleep to be impacted in participants with comorbid health conditions such as obstructive sleep apnea (4, 29, 30), oral health (31, 32), diabetes, cardiovascular disease (33–35), and mental health conditions (36). Furthermore, sex-differences in sleep health are well established and attributed in part due to life experiences (e.g., college years), hormonal changes (e.g., reproductive hormonal change, during menopause), and aging (37). As such, sex-differences in sleep health also contribute to biases in self-report of sleep (38, 39). Although our sample were predominantly female, no sex differences were reported in sleep-durations or between smartwatches which can be likely attributed to a relatively homogenous cohort in terms of age. Since the parent wellbeing studies did not assess comorbid health conditions such as diabetes or cardiovascular diseases, their impact on sleep durations measured using commercial-grade smartwatches warrants further research.

This study has limitations. This study was not designed with *a priori* sample size estimates to detect specific differences in reported versus measured sleep duration or quality. Reliance on self-reported sleep measures introduces potential recall bias despite instructions for timely completion. The wearable devices provide indirect measure of sleep based on actigraphy. This approach has the potential to misclassify periods of stillness as sleep or movement as wakefulness. Several factors not measurable by wearable devices also affect sleep quality, further complicating validation efforts. Furthermore, in the parent study on wellbeing, congenital health conditions were not assessed, which may affect sleep-related measurements and potentially introduce biases. Generalizability is limited by the short follow-up period and the unique sleep challenges faced by healthcare workers. Additionally, only one manufacturer’s family of devices (i.e., Garmin International) was included; future studies should evaluate sleep metrics from other manufacturers (e.g., Oura, Fitbit, Apple). Finally, although using self-report as a comparator is practical, it carries inherent limitations when compared to PSG.

## Conclusions

The growing sophistication of sensors in commercial-grade wearables and their rising global adoption by the population raises the possibility of increased adoption in biomedical research. This study’s findings suggest that wearable watches, especially newer generations (e.g. Venu 2 Plus and Venu 3/3S of Garmin International), can reliably estimate sleep duration, with strong concordance with self-reported data. On the other hand, their accuracy in assessing sleep quality was limited, with agreement observed primarily in the mid-range of sleep quality scores. Future research should expand validation efforts across various smartwatch models and manufacturers to assess the consistency of sleep duration and quality estimates.

## Data Availability

The datasets presented in this article are not readily available because the digital data was not approved/consented for public access due to privacy concerns and related challenges. Requests to access the datasets should be directed to chauhan.mohit@mayo.edu and athreya.arjun@mayo.edu.
