# Supplementary material for: The validation of measured and self-reported sleep duration and perceived sleep quality: an empirical study with three generations of smartwatches
Source: Front Psychiatry. 2026 Apr 20;17:1767216. doi: 10.3389/fpsyt.2026.1767216 (PMC13136088; doi:10.3389/fpsyt.2026.1767216)
Supplement: Supplementary file 1 [file Supplementaryfile1.docx]

**
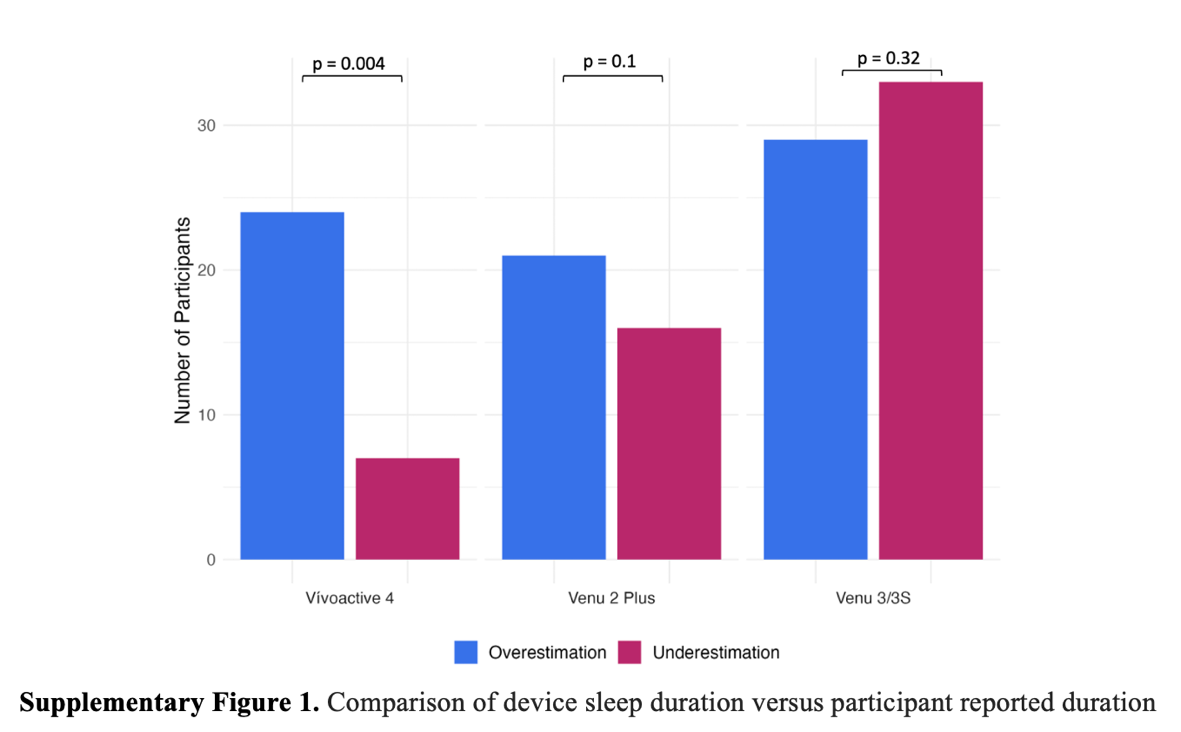
Supplementary FIGURES**

**Supplementary TABLES**

**Supplementary Table 1.** Results of ANOVA

| **Question** | **Sum of Squares** | **df** | **Mean Square** | **F** | **p-value** |
| --- | --- | --- | --- | --- | --- |
| **Q1** | 4362 | 3 | 1454.32 | 0.96 | 0.42 |
| **Q2** | 821 | 3 | 274 | 0.17 | 0.91 |
| **Q3** | 3166 | 4 | 792 | 0.51 | 0.73 |
| **Q4** | 3712 | 4 | 928 | 0.60 | 0.67 |
| **Q5** | 3743 | 3 | 1248 | 0.80 | 0.50 |
| **Q6** | 3543 | 3 | 1181 | 0.76 | 0.52 |
| **Q7** | 6029 | 2 | 3015 | 1.99 | 0.14 |
| **Q8** | 461 | 2 | 231 | 0.15 | 0.86 |
| **Q9** | 1389 | 3 | 463 | 0.30 | 0.83 |
| **Q10a** | 3971 | 3 | 1324 | 0.86 | 0.47 |
| **Q10b** | 13 | 2 | 7 | 0.004 | 0.99 |
| **Q10c** | 4248 | 3 | 1419 | 0.92 | 0.43 |
| **Q10d** | 1147 | 1 | 1147 | 0.73 | 0.39 |
| **Q10e** | 8106 | 3 | 2702 | 1.44 | 0.24 |
| **Q11** | 1120 | 3 | 373 | 0.24 | 0.87 |

**Supplementary Table 2.** Confusion matrix of Garmin sleep quality versus SQS

|  | **Excellent** | **Fair** | **Good** | **Poor** | **Terrible** |
| --- | --- | --- | --- | --- | --- |
| **Excellent** | 0 | 0 | 1 | 2 | 7 |
| **Fair** | 0 | 9 | 0 | 120 | 27 |
| **Good** | 1 | 7 | 9 | 95 | 137 |
| **Poor** | 0 | 0 | 0 | 15 | 5 |
| **Terrible** | 0 | 0 | 0 | 1 | 0 |

**Supplementary Table 3.** Global Sleep Assessment Questionnaire (GSAQ) question list

| **Question** | **Prompt** |
| --- | --- |
| Q1 | Did you have difficulty falling asleep. staying asleep, or feeling poorly rested in the morning? |
| Q2 | Did you fall asleep unintentionally or have to fight to stay awake during the day? |
| Q3 | Did sleep difficulties or daytime sleepiness interfere with your daily activities? |
| Q4 | Did work or other activities prevent you from getting enough sleep? |
| Q5 | Did you snore loudly? |
| Q6 | Did you hold your breath, have breathing pauses, or stop breathing in your sleep? |
| Q7 | Did you have restless or "crawling" feelings in your legs at night that went away if you moved  your legs? |
| Q8 | Did you have repeated rhythmic leg jerks or leg twitches during your sleep? |
| Q9 | Did you have nightmares, or did you scream, walk, punch, or kick in your sleep? |
| Q10a | Did the following things disturb your sleep: pain |
| Q10b | Did the following things disturb your sleep: other physical problems |
| Q10c | Did the following things disturb your sleep: worries |
| Q10d | 1 Did the following things disturb your sleep: medication |
| Q10e | Did the following things disturb your sleep: other |
| Q11 | Did you feel sad or anxious? |

**Supplementary Table 4.** Results of chi-squares comparing sleep quality categories

|  | **N** | **X squared** | **df** | **p-value** |
| --- | --- | --- | --- | --- |
| **Venu 2 Plus + Venus 3/3S** | 535 | 157.74 | 20 | <0.001 |
| **Venu 2 Plus** | 198 | 71.56 | 9 | <0.001 |
| **Venu 3/3S** | 337 | 110.7 | 20 | <0.001 |

**BROWNIE STUDY: SLEEP TIME VALIDATION and QUALITY ASSESSMENT**

**PIs: Mohit Chauhan, Arjun P. Athreya, and William V. Bobo**

| **NAME:** | **SHIFT TYPE**  **(TICK ONE)** | **DAY** | **NIGHT** | **FLEX** | **UNIT:** |
| --- | --- | --- | --- | --- | --- |

**INSTRUCTIONS:**

1. **Please ensure you wear your BROWNIE Study watch for the 7 days you log your sleep.**
2. **For Sleep Quality*:**
   1. *Please think about the quality of your sleep* ***overall****, such as how many hours of sleep you got, how easily you fell asleep, how often you woke up during the night (except to go to bathroom), how often you woke up earlier than you had to in the morning, and how refreshing your sleep was.*
   2. *SCORING SLEEP QUALITY:* ***Terrible – 0; Poor – 1-3; Fair – 4-6; Good – 7-9; Excellent – 10***

| **Day** | **Date going to Sleep**  **MM/DD/YYYY** | **Time going to Sleep**  **HH:MIN:AM/PM**  Ex.: 09:45:PM | **Date waking up from Sleep**  **MM/DD/YYYY** | **Time waking up from Sleep**  **HH:MIN:AM/PM**  Ex.: 09:45:AM | **On a scale of 1-10, Assess the Quality of Sleep**  **See instructions above** | **Enter Sleep Score and Quality from Garmin Connect App** | |
| --- | --- | --- | --- | --- | --- | --- | --- |
|  |  |  |  |  |  | **Score** | **Quality** |
| **1** |  |  |  |  |  |  |  |
| **2** |  |  |  |  |  |  |  |
| **3** |  |  |  |  |  |  |  |
| **4** |  |  |  |  |  |  |  |
| **5** |  |  |  |  |  |  |  |
| **6** |  |  |  |  |  |  |  |
| **7** |  |  |  |  |  |  |  |

**Supplementary Material 1.** Sleep diary given to participants
